# Association of electrochemical skin conductance with neuropathy in chemotherapy-treated patients

**DOI:** 10.1007/s10286-022-00895-w

**Published:** 2022-09-21

**Authors:** Fawaz Mayez Mahfouz, Susanna B. Park, Tiffany Li, Hannah C. Timmins, Lisa G. Horvath, Michelle Harrison, Peter Grimison, Tracy King, David Goldstein, David Mizrahi

**Affiliations:** 1 Brain and Mind Centre, School of Medical Sciences, Faculty of Medicine and Health, The University of Sydney, Camperdown, NSW 2050 Australia; 2 Chris O’Brien Lifehouse, Camperdown, NSW 2050 Australia; 3 Sydney Medical School, The University of Sydney, Camperdown, NSW 2050 Australia; 4 Royal Prince Alfred Hospital, Camperdown, NSW 2050 Australia; 5 Department of Medical Oncology, Liverpool Hospital, Liverpool, NSW 2170 Australia; 6 Cancer Nursing Research Unit, The University of Sydney, Camperdown, NSW 2050 Australia; 7 Institute of Haematology, Royal Prince Alfred Hospital, Camperdown, NSW 2050 Australia; 8 Prince of Wales Clinical School, Faculty of Medicine and Health, UNSW Sydney, Randwick, NSW 2031 Australia; 9 Department of Medical Oncology, Prince of Wales Hospital, Randwick, NSW 2031 Australia; 10 The Daffodil Centre, The University of Sydney, a joint venture with Cancer Council NSW, Camperdown, NSW 2050 Australia

**Keywords:** Chemotherapy-induced peripheral neuropathy (CIPN), Sudomotor, Sudoscan, Autonomic, Electrochemical skin conductance (ESC)

## Abstract

**Purpose:**

Chemotherapy-induced peripheral neuropathy (CIPN) is an adverse event of cancer treatment that can affect sensory, motor, or autonomic nerves. Assessment of autonomic neuropathy is challenging, with limited available tools. Accordingly, it is not routinely assessed in chemotherapy-treated patients. In this study, we aimed to examine whether electrochemical skin conductance (ESC) via Sudoscan, a potential measure of autonomic function, associates with subjective and objective measures of CIPN severity and autonomic neuropathy.

**Methods:**

A cross-sectional assessment of patients who completed neurotoxic chemotherapy 3–24 months prior was undertaken using CIPN patient-reported outcomes (EORTC-QLQ-CIPN20), clinically graded scale (NCI-CTCAE), neurological examination score (TNSc), autonomic outcome measure (SAS), and Sudoscan. Differences in CIPN severity between participants with or without ESC dysfunction were investigated. Linear regression analyses were used to identify whether ESC values could predict CIPN severity.

**Results:**

A total of 130 participants were assessed, with 93 participants classified with CIPN according to the clinically graded scale (NCI-CTCAE/grade ≥ 1), while 49% demonstrated hands or feet ESC dysfunction (*n* = 46). Participants with ESC dysfunction did not significantly differ from those with no dysfunction on multiple CIPN severity measures (clinical-grade, patient-report, neurological examination), and no differences on the autonomic outcome measure (SAS) (all *p* > 0.0063). Linear regression analyses showed that CIPN could not be predicted by ESC values.

**Conclusions:**

The inability of ESC values via Sudoscan to predict clinically-graded and patient-reported CIPN or autonomic dysfunction questions its clinical utility for chemotherapy-treated patients. The understanding of autonomic neuropathy with chemotherapy treatment remains limited and must be addressed to improve quality of life in cancer survivors.

## Introduction

Chemotherapy-induced peripheral neuropathy (CIPN) is a common adverse effect of numerous neurotoxic chemotherapy agents, including vinca alkaloids, taxanes, platinum compounds, bortezomib, and thalidomide [1]. The pathophysiological mechanisms of CIPN remain ill-defined; however, off-target effects of chemotherapy on peripheral nerve fibres may trigger its manifestation in patients [2]. CIPN produces symptoms of sensory loss, paraesthesia, poor dexterity, and pain, which may significantly impact the patient’s quality of life [3]. Although CIPN predominantly affects large sensory nerve fibre function [4], small sensory fibres may also be affected, resulting from damage occurring to unmyelinated C-fibres and thinly myelinated A-delta fibres [5]. Small fibre neuropathy may be accompanied by symptoms of sporadic burning and shock-like pain [6], as well as impairment of autonomic function [7], including blood pressure, digestion and perspiration [8].

While there remains no gold-standard clinical outcome measures for CIPN [9], there are a range of validated methods examining large fibre dysfunction in CIPN, including neurophysiological assessments, clinical examination [10] and patient-reported outcome measures [11]. However, there is a lack of validated tools to measure small nerve fibre damage or autonomic neuropathy in CIPN [12]. Available techniques to examine small nerve fibre integrity, such as skin biopsy, have limited utility in clinical practice due to invasiveness, cost and delays in receiving results [13].

Since sudomotor sweat gland function is innervated by small nerve fibres [14], techniques have been developed to assess sudomotor function to provide an index of autonomic neuropathy. The Quantitative Sudomotor Axon Reflex Test (QSART) is a sensitive and reproducible test of sudomotor function, but is limited by cost and extensive patient preparation time [15, 16]. Since electrochemical skin conductance (ESC) depends on sweat gland function [17], its values may quantify sudomotor function and provide a surrogate marker for autonomic neuropathy. Sudoscan has been developed as a method to measure ESC, with preliminary findings suggesting its potential use as a measure of small nerve fibre function across disorders, such as diabetic peripheral neuropathy [16]. Nevertheless, the Sudoscan technique has been criticised as lacking evidence for a direct link between ESC and small nerve fibre or autonomic function, as well as discrepancies with normative datasets [18]. A blinded-prospective study demonstrated reduced intraepidermal nerve fibre density (IENFD) measured via skin biopsy was associated with lower ESC values via Sudoscan in small fibre neuropathy [19]. However, a subsequent cohort study of patients with polyneuropathy found that the association between ESC and IENFD was not strong, and that additional mechanisms may be required to explain sweat gland dysfunction in peripheral neuropathy [20].

Sudoscan techniques have only been utilised in three previous CIPN studies [15, 21, 22]. Although reduced ESC values were associated with CIPN severity [22], including the Total Neuropathy Score [15] and measures of neuropathic pain [21], broader comparisons of CIPN outcome measures and ESC values in patients with CIPN are needed to investigate the utility of Sudoscan in this population. Further, mechanistic understanding of the physiological contributors to ESC values are needed to determine the clinical significance of reduced ESC in the context of CIPN.

Therefore, the primary aim of this study was to examine the association of ESC dysfunction with clinical, patient-reported, and neurophysiological measures of CIPN among neurotoxic chemotherapy-treated patients. Additionally, we aimed to identify whether ESC values via Sudoscan were predictive of CIPN severity, pain, and autonomic outcomes.

## Methods

### Participants

Participants who completed neurotoxic chemotherapy (including taxanes, platinum-based agents, bortezomib, vinca alkaloids and thalidomide) were recruited cross-sectionally from Sydney, Australia, between June 2017 and March 2020. Participants who were aged ≥ 18 years and 3–24 months post-treatment were eligible. The study was approved by Sydney Local Health District (RPAH zone) Human Research Ethics Committee, with informed consent obtained from each participant.

### Procedures

Clinical data (age, height, chemotherapy type, cancer diagnosis and stage) were retrieved from medical records. Participants’ weight was assessed during their study visit. Body mass index (BMI) was calculated as kg/m^2^.

### Electrochemical skin conductance measurement

ESC was evaluated by assessing sweat gland function using the Sudoscan device (Impeto Medical, Paris, France) [23]. Participants placed their palms (hands) and soles (feet) onto the electrodes in a standing position for 2–3 min. A direct current of ≤ 4 V was applied through the electrodes by chronoamperometry which generated a current relative to the chloride ions extracted from the skin through the mechanism of reverse iontophoresis [23–27]. The ESC values were quantified in microSiemens (µS) based on the reaction between chloride ions from the sweat glands and the direct current generated from the electrodes [23]. The electrodes were sterilised before each test, and the test was repeated twice for both the hands and feet, with the average ESC value taken separately for the hands and feet. Average ESC values were categorised as no dysfunction (≥ 60 μS) or dysfunction (< 60 μS), as in prior studies [24, 26]. Participants were classified with ESC dysfunction if they had dysfunction in the hands, feet, or both.

### Clinical neuropathy assessment

CIPN severity was assessed using the Total Neuropathy Score-clinical version (TNSc©, Johns Hopkins University), a composite tool of six domains including upper and lower limb pin-prick sensory and vibration sensibility, deep tendon reflexes, strength assessment and patient-reported sensory and motor symptoms [28, 29]. Each domain was graded between 0 ‘normal’ and 4 ‘severe’, with a total score ranging from 0 ‘no neuropathy’ to 24 ‘severe neuropathy’; Researchers completed training to ensure reliability across assessors. Researchers graded CIPN severity via the National Cancer Institute Common Terminology Criteria for Adverse Events (NCI-CTCAE) sensory neuropathy subscale Version 4.0 categorised: Grade 0 ‘no symptoms’, Grade 1 ‘asymptomatic, not interfering with daily function’, Grade 2 ‘moderate symptoms, limiting daily function’, Grade 3 ‘severe symptoms, limiting daily function and self-care’, and Grade 4 ‘disabling’[30]. Nerve conduction studies (NCS) were undertaken to record maximal amplitude of lower limb tibial and sural nerves, using methodology as reported in previous studies [31].

### Patient-reported outcome measures

The European Organisation of Research and Treatment of Cancer Quality of Life Questionnaire-Core (EORTC-QLQ-CIPN-20) is a 20-item validated questionnaire assessing motor, sensory and autonomic peripheral neuropathy symptoms, rating each item on a 4-point Likert scale from 1 ‘not at all’ to 4 ‘very much’, converted to a 0–100 scale, with higher scores indicating more severe CIPN [32]. The Survey of Autonomic Symptoms (SAS) questionnaire is an 11-item questionnaire to measure autonomic symptoms based on two scores: total number of symptoms (SAS symptom score), and total impact score (SAS impact score) graded from 1 ‘least severe’ to 5 ‘most severe’ for each reported symptom [33]. Questions assessing the following autonomic symptom domains were grouped: sudomotor, gastrointestinal, vasomotor, orthostatic, and urinary function. The total number of symptoms is calculated as the sum of total reported symptoms, whilst the total impact score is the sum of the total burden from each reported symptom. The SAS domains have been well-validated with other measures of autonomic function, displaying strong correlations with Autonomic Symptom Profile (ASP) domains and QSART measurements [33]. Male specific questions (Question 20 EORTC-QLQ-CIPN20; question 12 SAS) were omitted from analysis. The Pain Numeric Rating Scale (PNRS) was utilised to assess the worst neuropathic pain that participants have experienced in the last 24 h prior to testing. The scale ranges from 0 to 10, with ‘0’ representing ‘no pain at all’ and 10 representing ‘the worst pain possible’ [34].

### Statistical analyses

All analyses were conducted using SPSS Statistics Software V27 (IBM; Armonk, NY, USA) and followed the STROBE statement for observational studies [35]. Normality of data was assessed using the Shapiro–Wilk test. Normally distributed data (*p* > 0.05) were presented as mean ± standard deviation (SD), while non-normally distributed data (*p* < 0.05) were presented as medians and interquartile range (IQR). Independent sample *t* tests or Mann–Whitney *U* tests were used for normally and non-normally distributed data, respectively, to explore mean differences between clinical, neurophysiological and CIPN outcome measure scores of the two cohorts (participants with ESC dysfunction vs. no-ESC dysfunction). The associations between ESC values via Sudoscan, clinical characteristics, CIPN, pain and autonomic outcome measures were calculated using Pearson’s or Spearman’s correlation coefficients for normally and non-normally distributed data, respectively. Where specified, the Bonferroni correction was applied, modifying the significance level from *p* < 0.05 to *p* < 0.0063 due to the number of contrasts. Finally, we examined the ability of ESC values and clinical characteristics to predict patient scores on CIPN severity and autonomic outcome measures using linear regression. The independent variables were age, sex, BMI, hand ESC and feet ESC. Dependent variables were scores of patient-reported outcome measure (EORTC-QLQ-CIPN20), neurological examination score (TNSc), sural and tibial amplitudes, and sudomotor dysfunction of the autonomic outcome measure (SAS). The independent variables of the model were checked for multicollinearity. Linear regression was bootstrapped to account for non-normal distribution of the residuals and to produce robust confidence intervals.

## Results

### Demographics and clinical history

A total of 130 neurotoxic chemotherapy-treated participants were assessed cross-sectionally at a median of 6.0 (3.0–12.0) months post-treatment completion. Of these, 67% were female (*n* = 87), and the median age at the time of assessment was 58.6 years (47.6–66.5) (Table 1). The most common cancer types were breast (33%, *n* = 43) and gynaecological (21%, *n* = 27). The most common chemotherapy types were taxanes (61%, *n* = 79) and platinum-based (24%, *n* = 31). Clinical and demographic information is displayed in Table 1.

**Table 1:** Clinical and demographic characteristics of participants

	Total cohort (*n* = 130)	
	*n*	%
Sex, female	87	67
Cancer types		
Breast	43	33
Gynaecological	27	21
Haematological	19	15
GI/colorectal	14	11
Testicular	12	9
Other (prostate, pancreatic and urothelial)	15	11
Chemotherapy types		
Taxane	79	61
Platinum-based	31	24
Bortezomib	17	13
Thalidomide	2	1.5
Nab-paclitaxel	1	0.5
Cancer stage of solid tumours		
I	8	6
II	28	22
III	34	26
IV	38	29
Non-solid (no stage)	19	15
Undefined	3	2
	Median	IQR (25th –75th percentile)
Age (years)	58.6	47.6–66.5
BMI (kg/m^2^)	27.1	23.8–30.6
Months since treatment completion	6.0	3.0–12.0

### Chemotherapy-induced peripheral neuropathy profile

Overall, 28% of participants (*n* = 37) had no CIPN symptoms (Grade-0) at the time of assessment using a clinically graded scale (NCI-CTCAE), while 72% (*n* = 93) were graded with CIPN symptoms of any severity (Grade ≥ 1), while 23% were classified with mild CIPN (Grade-1; *n* = 30), 42% with moderate (Grade-2, *n* = 54), and 7% with severe CIPN (Grade-3, *n* = 9). Using the neurological examination score (TNSc), the median score of the cohort was 3.5 (2.0–6.0) (out of 24). From the TNSc score, 62% had reduced pinprick sensation (score ≥ 1, *n* = 81), 22% had reduced vibration sensation (score ≥ 1, *n* = 29), 73% had abnormal tendon reflexes (score ≥ 1, *n* = 95) and none had reduced ankle plantar flexor strength (score = 0, *n* = 130). Also, 17% (*n* = 22) reported some nerve pain (≥ 1/10) in the 24 h prior to the study visit. Based on the patient-reported autonomic neuropathy outcome measure (SAS), completed by 81 participants, 52% reported having sudomotor dysfunction (*n* = 42), 45% reported vasomotor dysfunction (*n* = 36), 36% reported orthostatic dysfunction (*n* = 29), 28% reported gastrointestinal dysfunction (*n* = 20) and 11% reported urinary dysfunction (*n* = 9).

Participants with CIPN were older (*p* < 0.001) and had significantly greater CIPN severity score on multiple CIPN outcome measures versus those without CIPN (Table 2), including the patient-reported outcome (EORTC-QLQ-CIPN20; *p* < 0.001), clinically graded scale (NCI-CTCAE; *p* < 0.001) and neurological examination scores (TNSc; *p* < 0.001). Sural and tibial amplitudes were significantly reduced in participants with CIPN compared to those without CIPN (all *p* < 0.002) (Table 2). In patients with CIPN, higher scores on the patient-reported outcome measure (EORTC-QLQ-CIPN20) correlated with higher autonomic outcome measure (SAS) symptom score (*r* = 0.48) and total impact score (*r* = 0.47) (both *p* < 0.001). However, despite this, there was no significant difference in the autonomic outcome measure (SAS) domain scores between patients with and without CIPN (all *p* > 0.0063) (Table 2). ESC values via Sudoscan, including average hand ESC and feet ESC, were also not statistically different between participants with or without CIPN (all *p* > 0.0063) (Table 2).

**Table 2:** Comparison of neuropathy outcomes between patients with ESC and no ESC dysfunction

Assessment tools	No CIPN (NCI-CTCAE grade 0) (*n* = 37)		CIPN (NCI-CTCAE grade ≥ 1) (*n* = 93)		*P* value
	Median	IQR (25–75th percentile)	Median	IQR (25–75th percentile)	
Clinical characteristics					
Age (years)	**49**	**35.7–55.2**	**61.1**	**53.5–68.6**	** < 0.001**
BMI (kg/m^2^)	26.4	22.6–30.5	27.1	23.8–30.6	0.40
CIPN outcome measures					
EORTC-QLQ-CIPN20	**0**	**0–1.8**	**14.0**	**8.8–22.8**	** < 0.001**
NCI-CTCAE	**0**	**0–0**	**2**	**1–2**	** < 0.001**
TNSc	**1**	**0–3**	**5**	**3–7**	** < 0.001**
Neurophysiological measurements					
Tibial amplitudes (mV), mean (SD)*	**12.7**	**4.6**	**9.7**	**4.3**	**0.002**
Sural amplitudes (µV)	**18**	**10.3–22.0**	**7.5**	**4.5–12.0**	** < 0.001**
Pain measures					
PNRS	0	0–0	0	0–0	0.02
Autonomic outcome measures					
Symptom score	1	0–2	2	1.0–3.3	0.02
Total impact score	1	0–5	4	1–8	0.03
Orthostatic dysfunction	0	0–0	0	0–1	0.29
Sudomotor dysfunction	0	0–1	1	0–1	0.03
Vasomotor dysfunction	0	0–1	1	0–1	0.04
Gastrointestinal dysfunction	0	0–0	0	0–1	0.42
Urinary dysfunction	0	0–0	0	0–0	0.67
Electrochemical skin conductance via sudoscan					
Hands ESC (average)	70	60.8–78.0	66	51.8–73.5	0.17
Feet ESC (average)	74.5	69.5–79.8	71.0	56.3–78.3	0.04

*p* < 0.0063 was considered significant due to Bonferroni correction (in bold)

*Indicates *p* values using independent sample *t* tests

### ESC dysfunction and CIPN severity in cancer survivors exposed to neurotoxic chemotherapy

**Fig. 1:**
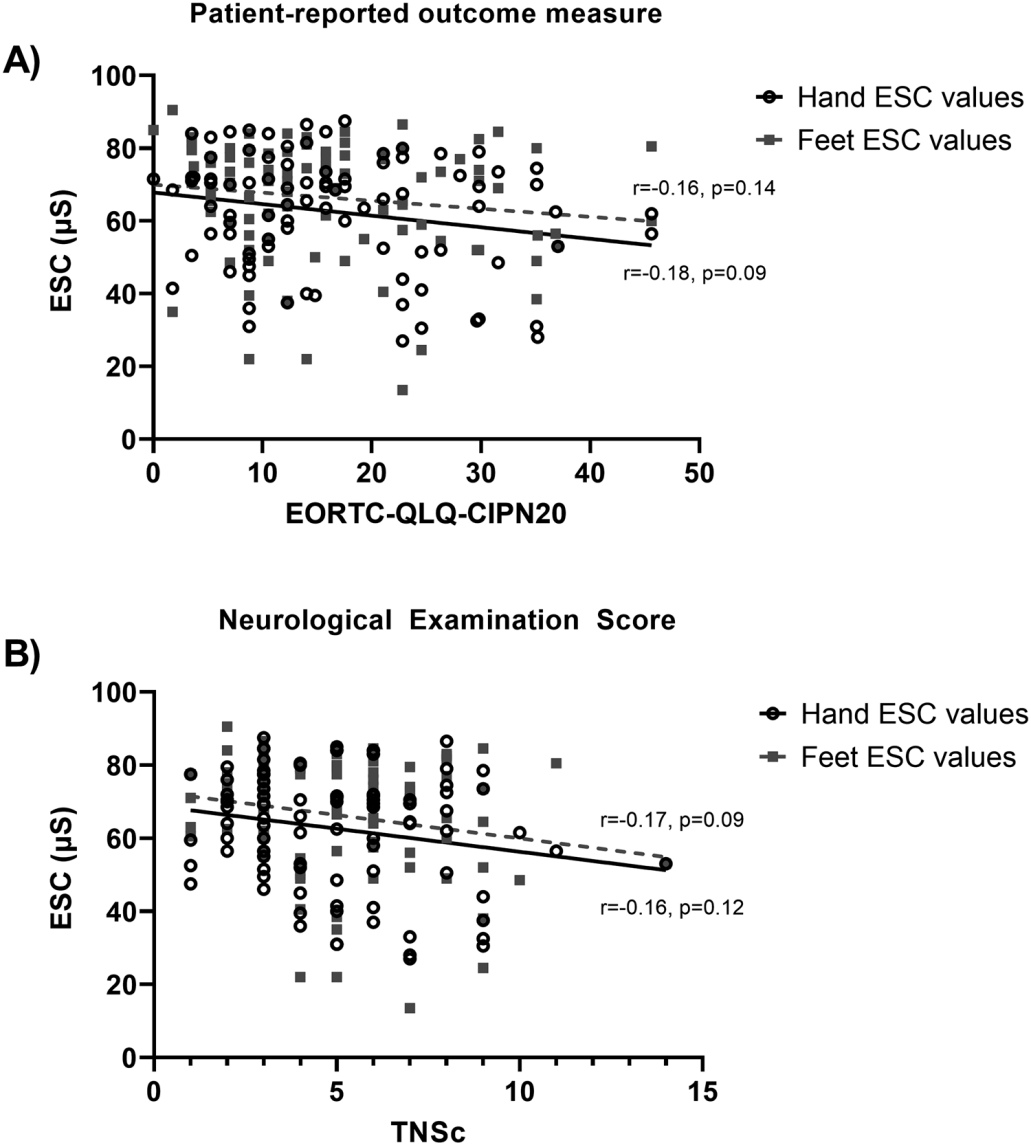
ESC values (via Sudoscan) of the hands and feet with **A** patient-reported outcome measure (EORTC-QLQ-CIPN20) and **B** neurological examination score (TNSc); *solid line* the line of best fit for hand ESC, and *dashed line* feet ESC

Of the 93 participants with CIPN, 49% (*n* = 46) had any ESC dysfunction, while 51% (*n* = 47) had no dysfunction, and 39% (*n* = 36) experienced ESC dysfunction in their hands, 30% (*n* = 28) experienced ESC dysfunction in their feet, while 19% (*n* = 18) had dysfunction in both their hands and feet. There were no significant correlations between clinical, neurophysiological, or autonomic outcome measures with ESC values for either hands or feet (all *p* > 0.0063) (Table 3), including the patient-reported outcome measure (EORTC-QLQ-CIPN20) and the neurological examination score (TNSc) (Fig. 1).

**Table 3:** Associations between clinical characteristics, neurophysiological measurements and CIPN outcome measures with hands and feet ESC in participants with CIPN

Assessment tools	Hands ESC (*n* = 93)	Feet ESC (*n* = 93)
Clinical characteristics		
Age (years)	*r*_s_ = − 0.19, *p* = 0.06	*r*_s_ = − 0.25, *p* = 0.02
BMI (kg/m^2^)	*r*_s_ = 0.14, *p* = 0.19	*r*_s_ = − 0.02, *p* = 0.88
Neurophysiological measurements		
Tibial amplitudes (mV)*	*r* = 0.15, *p* = 0.20	*r* = 0.15, *p* = 0.21
Sural amplitudes (µV)	*r*_s_ = 0.14, *p* = 0.23	*r*_s_ = 0.24, *p* = 0.04
CIPN outcome measures		
TNSc	*r*_s_ = − 0.16, *p* = 0.12	*r*_s_ = − 0.17, *p* = 0.09
EORTC-QLQ-CIPN 20	*r*_s_ = − 0.18, *p* = 0.09	*r*_s_ = − 0.16, *p* = 0.14
NCI-CTCAE	*r*_s_ = − 0.19, *p* = 0.07	*r*_s_ = − 0.17, *p* = 0.09
Pain measures		
PNRS	*r*_s_ = 0.01, *p* = 0.96	*r*_s_ = − 0.21, *p* = 0.05
Autonomic outcome measures		
Symptom score	*r*_s_ = 0.02, *p* = 0.91	*r*_s_ = − 0.12, *p* = 0.36
Total impact score	*r*_s_ = − 0.04, *p* = 0.79	*r*_s_ = − 0.12, *p* = 0.35
Sudomotor dysfunction	*r*_s_ = − 0.09, *p* = 0.49	*r*_s_ = − 0.20, *p* = 0.13
Vasomotor dysfunction	*r*_s_ = − 0.10, *p* = 0.48	*r*_s_ = − 0.06, *p* = 0.66
Gastrointestinal dysfunction	*r*_s_ = 0.01, *p* = 0.97	*r*_s_ = − 0.14, *p* = 0.31
Urinary dysfunction	*r*_s_ = 0.04, *p* = 0.79	*r*_s_ = 0.02, *p* = 0.87

*p* < 0.0063 was considered significant due to Bonferroni correction. The use of Pearson’s *r* or Spearman’s *r*_s_ is denoted in the table

CIPN severity from patient-reported outcome (EORTC-QLQ-CIPN20), clinically graded scale (NCI-CTCAE) and neurological examination score (TNSc) were not significantly different between participants with and without ESC dysfunction (all *p* > 0.0063) (Table 4). Neurophysiological measurements did not significantly differ between participants with and without ESC dysfunction (*p* > 0.0063). None of the individual items or total scores of the autonomic outcome measure were different between participants with and without ESC dysfunction (*p* > 0.0063) (Table 4).

**Table 4:** Comparison of neuropathy outcomes between CIPN participants (NCI-CTCAE ≥ 1, *n* = 93) with ESC and no ESC dysfunction

Assessment tools	ESC dysfunction (*n* = 46)		No ESC dysfunction (*n* = 47)		*P* value
	Median	IQR (25–75th percentile)	Median	IQR (25–75th percentile)	
CIPN outcome measures					
EORTC-QLQ-CIPN20	16.2	8.8–27.1	14.0	5.3–17.5	0.07
NCI-CTCAE	2	1.75–2	2	1–2	0.02
TNSc	5	3–7	4	3–6	0.16
Clinical characteristics					
Age (years)	62.2	57.0–67.7	60.3	50.8–69.3	0.45
BMI (kg/m^2^)	26.9	22.8–30.7	27.2	24.3–30.6	0.45
Neurophysiological measurements					
Tibial amplitudes (mV), mean (SD)*	8.7	3.9	10.6	4.4	0.06
Sural amplitudes (µV)	7.3	3.4–11.1	8	5.5–13.3	0.10
Pain measures					
PNRS	0	0–3	0	0–0	0.12
Autonomic outcome measures					
Symptom score	2	0.5–4.5	2	1.5–3.0	0.79
Total impact score	5	0.5–8.5	4	2.0–7.5	0.86
Orthostatic dysfunction	0	0–1	0	0–1	0.43
Sudomotor dysfunction	1	0–1	0	0–1	0.23
Vasomotor dysfunction	1	0–1	1	0–1	0.90
Gastrointestinal dysfunction	0	0–1	0	0–1	0.95
Urinary dysfunction	0	0–0	0	0–0	0.23

*p* < 0.0063 was considered significant due to Bonferroni correction

*Indicates *p* values using independent sample *t* tests

### Predictive models of CIPN severity

Linear regression analyses revealed that age was a significant predictor of all clinically graded and patient-reported CIPN severity measures, including the patient-reported outcome (EORTC-QLQ-CIPN20, *p* = 0.002) and the neurological examination score (TNSc, *p* = 0.001), but not of patient-reported sudomotor function (*p* > 0.05) (Table 5). Sex was a predictor of sural amplitudes (*p* = 0.001), while BMI was a predictor of tibial amplitudes (*p* = 0.003) (Table 5). Neither hand ESC nor feet ESC values could predict CIPN severity with any measures, including the sudomotor dysfunction sub-scale of the autonomic outcome measure (all *p* > 0.05) (Table 5).

**Table 5:** Linear regression analyses of Sudoscan ESC values and clinical characteristics to predict CIPN severity, neurophysiological outcomes or sudomotor dysfunction

Dependent variable	Independent variables	Parameter estimate [95% confidence interval]	*P* value
CIPN outcome measures			
EORTC-QLQ-CIPN20	Age	**0.24 [0.09, 0.40]**	**0.002**
	Sex	0.04 [− 4.6, 3.9]	0.99
	BMI	− 0.02 [− 0.4, 0.4]	0.93
	Hand ESC	− 0.13 [− 0.31, 0.04]	0.12
	Feet ESC	− 0.02 [− 0.21, 0.17]	0.82
TNSc	Age	**0.11 [0.08, 0.13]**	**0.001**
	Sex	− 0.73 [− 1.6, 0.11]	0.10
	BMI	− 0.009 [− 0.09, 0.08]	0.79
	Hand ESC	− 0.02 [− 0.06, 0.02]	0.39
	Feet ESC	− 0.009 [− 0.05, 0.02]	0.61
Neurophysiological measurements			
Sural amplitudes	Age	− **0.26 [**− **0.41, **− **0.12]**	**0.001**
	Sex	**6.2 [3.06, 9.47]**	**0.001**
	BMI	− 0.04 [− 0.38, 0.27]	0.83
	Hand ESC	− 0.16 [− 0.38, 0.07]	0.12
	Feet ESC	0.14 [− 0.01, 0.27]	0.07
Tibial amplitudes	Age	− **0.14** **[**− **0.20,** − **0.08]**	**0.001**
	Sex	1.48 [− 0.23, 3.26]	0.10
	BMI	− **0.18** **[**− **0.33, 0.0002]**	**0.03**
	Hand ESC	0.07 [− 0.01, 0.16]	0.10
	Feet ESC	− 0.04 [− 0.12, 0.03]	0.28
Autonomic outcome measure			
SAS–sudomotor dysfunction	Age	− 0.005 [− 0.02, 0.006]	0.37
	Sex	0.34 [− 0.10, 0.70]	0.07
	BMI	− 0.003 [− 0.03, 0.02]	0.84
	Hand ESC	0.001 [− 0.02, 0.02]	0.92
	Feet ESC	− 0.01 [− 0.03, 0.008]	0.33

*p* < 0.05 indicates statistical significance (in bold)

## Discussion

There is a need to establish reliable and easily implementable measures of nerve dysfunction among patients treated with neurotoxic chemotherapy. In particular, assessment of autonomic neuropathy in the context of CIPN has been inadequately explored. This study investigated an easily implementable measure of autonomic and small nerve fibre neuropathy associated with patient-reported and clinical measures of CIPN severity. However, hands and feet Sudoscan ESC values were not associated with CIPN measures nor autonomic outcome measures. Furthermore, ESC values failed to predict CIPN severity or autonomic neuropathy using linear regression analyses.

While there are a range of assessment tools for large fibre neuropathy in chemotherapy-treated patients, assessment of small fibre neuropathy and autonomic dysfunction remains limited [12]. IENFD, assessed via skin biopsy, provides a diagnostic tool for small fibre neuropathy. However, while some studies have revealed reduced IENFD with neurotoxic chemotherapy, others have not found reduced IENFD following treatment [12, 36]. Further, routine use of skin biopsy in clinical settings is not practical. Accordingly, other methods have been developed to attempt to assess small nerve fibre integrity and autonomic function. These include measurement of sudomotor activity via ESC as a measure of electrically-induced chloride ion conductance from the sweat glands on the skin surface [23]. However, it remains unclear if ESC reflects sudomotor fibre activity directly or is largely a measure of sweat gland activity [37].

Despite this lack of consensus, reduced ESC values have been found across a range of peripheral neuropathies, particularly in diabetic neuropathy [38]. Similarly, multiple studies have identified reduced ESC in hands and feet in chemotherapy-treated patients [15, 21, 22]. In agreement with these studies, we found evidence of reduced ESC values in a large proportion of our CIPN cohort; however, reduced ESC values were not associated with any CIPN outcome measures. Furthermore, ESC values were not predictive of CIPN severity or autonomic function using linear regression analyses. Accordingly, our findings do not provide support for the utility of ESC measurement as a diagnostic tool in patients with established CIPN. In contrast, Saad et al. [15] examined longitudinal change in ESC values during neurotoxic chemotherapy treatment, but did not examine the long-term effect of chronic CIPN on ESC values, as in the present study. Two smaller studies have demonstrated a link between CIPN severity and ESC values in 18 bortezomib-treated patients [22] and pain severity and reduced hands and feet ESC values in 36 oxaliplatin-treated patients [21]. In contrast, the current study showed no association of pain symptoms with reduced or increased hands or feet ESC values. Accordingly, the findings of these previous studies do not align with the results identified in the current study.

The inability for ESC values to accurately predict clinically-graded and patient-reported CIPN severity and autonomic function may relate to a lack of specificity in the ESC measurement. Initially, ESC values were used as an assessment of sweat function [23–27]. Gradually, it transitioned into a measure of sudomotor function [23–27], and finally into a measure of autonomic and small nerve fibre function in patients with underlying medical conditions, such as diabetes [38] and cystic fibrosis [23–27]. However, there remains a lack of evidence for a direct link between ESC and small nerve fibre function, as well as discrepancies with normative datasets, which greatly limit its clinical utility [18].

Overall, this study used a range of methods to measure CIPN severity, including patient-reported, clinically-graded, objectively measured, and neurophysiological measures. However, we did not have access to more objective quantification of autonomic or small nerve fibre neuropathy, such as skin biopsies, QSART [13] or autonomic reflex screen, and assessed autonomic neuropathy via a subjective patient-reported questionnaire. In our study, neither the autonomic outcome measure total score nor sub-scale scores were associated with ESC values. However, the SAS is a subjective tool for quantifying autonomic dysfunction and may be limited in this context due to the overlap between symptoms of CIPN and other effects of cancer and its treatment. Furthermore, the cross-sectional study design did not allow for examination of changes in ESC values and CIPN severity over time, including accounting for pre-treatment values. Additionally, our sample included a range of different cancer and treatment types which makes it challenging to determine if there were specific patterns of ESC changes in particular patient cohorts.

## Conclusion

ESC values measured by Sudoscan were not associated with CIPN severity using multiple outcome measures, and were not associated with patient-reported nor autonomic neuropathy measures. The discrepancies in the findings of prior studies and the inability of ESC values to predict clinically-graded and patient-reported CIPN or autonomic dysfunction may limit its utility in the clinic for assessing chemotherapy-treated patients. The results of our study highlight the need for a better measure of small nerve fibre and autonomic neuropathy with greater sensitivity in the context of CIPN. Understanding the CIPN phenotype may inform appropriate treatment strategies to reduce neuropathy burden and promote a better quality of life for affected patients.

## Data Availability

The datasets generated during and analysed during the current study are available from the corresponding author on reasonable request.
